# Embelin downregulated cFLIP in breast cancer cell lines facilitate anti-tumor effect of IL-1β-stimulated human umbilical cord mesenchymal stem cells

**DOI:** 10.1038/s41598-021-94006-w

**Published:** 2021-07-19

**Authors:** Ya-Han Liang, Jiann-Ming Wu, Jui-Wen Teng, Eric Hung, Hwai-Shi Wang

**Affiliations:** 1 Department of Anatomy, Institute of Anatomy and Cell Biology, School of Medicine, National Yang Ming Chiao Tung University, Peitou, Taipei, 112 Taiwan, ROC; 2 General Surgery Division, Far Eastern Memorial Hospital, New Taipei City, Taiwan, ROC

**Keywords:** Breast cancer, Cancer, Cell biology, Cell death

## Abstract

Breast cancer is the leading cause of cancer-related death for women. In breast cancer treatment, targeted therapy would be more effective and less harmful than radiotherapy or systemic chemotherapy. Tumor necrosis factor-related apoptosis-inducing ligand (TRAIL) has been shown to induce apoptosis in cancer cells but not in normal cells. Mesenchymal stem cells have shown great therapeutic potential in cancer therapy owing to their ability of homing to tumor sites and secreting many kinds of anti-tumor proteins including TRAIL. In this study, we found that IL-1β-stimulated human umbilical cord-derived mesenchymal stem cells (hUCMSCs) enhance the expression of membrane-bound and soluble TRAIL. Cellular FADD-like IL-1β-converting enzyme inhibitory protein (cFLIP) is an important regulator in TRAIL-mediated apoptosis and relates to TRAIL resistance in cancer cells. Previous studies have shown that embelin, which is extracted from *Embelia ribes*, can increase the TRAIL sensitivity of cancer cells by reducing cFLIP expression. Here we have demonstrated that cFLIP_L_ is correlated with TRAIL-resistance and that embelin effectively downregulates cFLIP_L_ in breast cancer cells. Moreover, co-culture of IL-1β-stimulated hUCMSCs with embelin-treated breast cancer cells could effectively induce apoptosis in breast cancer cells. The combined effects of embelin and IL-1β-stimulated hUCMSCs may provide a new therapeutic strategy for breast cancer therapy.

## Introduction

According to the World Health Organization (WHO) report, breast cancer is the most common cancer in women and also the leading cause of cancer-related death in women^1^. There are many types of breast cancer cells including the most common forms, ductal carcinoma in situ (DCIS) and invasive carcinoma. Surgery is a general therapy for breast cancer and is often combined with radiotherapy and chemotherapy to avoid the recurrence of breast cancer. It has been found that breast cancer patients suffer more pain due to complications and inflammation after radiotherapy and chemotherapy^2^. Owing to the recurrence of breast cancer, hormone therapy and targeted therapy were developed^3,4^. Targeted therapies are less harmful to normal cells. TNF-related apoptosis-inducing ligand (TRAIL) has been reported to induce apoptosis in different tumor cell lines including breast cancer but not normal cells^5,6^. Therefore, TRAIL-targeted therapy has attracted significant attention in cancer treatment.

Mesenchymal stem cells (MSCs) are fibroblast-like multipotent cells with self-renewal ability. MSCs can be isolated from many tissues such as adipose, bone marrow, and umbilical cord tissue. They are capable of differentiating into a variety of different cell types^7^. MSCs have shown enormous potential in treatment of different diseases owing to their homing ability, immune modulation, and protein secretion^8^. Several results have indicated that human bone marrow-derived mesenchymal stem cells (hBMMSCs) could suppress tumor growth, but other studies show that hBMMSCs could also be recruited to tumor site where they transform into tumor-associated fibroblasts (TAFs) which then enhance tumor growth^9^. Unlike hBMMSCs, human umbilical cord-derived mesenchymal stem cells (hUCMSCs) did not transform to TAFs in the presence of breast and ovarian cancer^9^. In addition to the ability to be easily isolated, hUCMSCs also hold an advantage over hBMMSCs in tumor suppression. It has been found that the growth of breast cancer cells can be attenuated by naïve hUCMSCs^10^. According to these findings, hUCMSCs are more suitable for cell therapy^11^.

Many reports demonstrated that microvesicles derived from MSCs induced cell apoptosis and cell cycle arrest^12,13^. MSCs have been engineered as a transmitter to deliver anti-tumor proteins like interferons, interleukins and soluble tumor necrosis factor-related apoptosis-inducing ligands (soluble TRAIL)^14–16^. TNF-related apoptosis-inducing ligand (TRAIL), also known as Apo-2 ligand, is a protein belonging to the tumor necrosis factor (TNF) ligand family. Most of the normal cells express membrane form TRAIL and also secrete soluble form TRAIL. Previous studies have shown that TRAIL could induce cell apoptosis in many kinds of cancer cells^17,18^.

Apoptosis has two main pathways: the intrinsic and extrinsic pathways. The intrinsic pathway is initiated through cell stress or damage and is controlled by the Bcl 2 family. Regulation of apoptosis occurs through perforation of the mitochondrial membrane to release cytochrome c into the cytoplasm and combine with pro-caspase-9 to form the apoptosome. Then the apoptosome activated caspase-3 leads to cell destruction^19^. The extrinsic way is triggered by ligands like Fas and TRAIL binding to cell surface death receptors. The binding of ligand and receptor changes the Fas-associated death domain protein (FADD) to activate caspase-8/10 and caspase-3^20^. Both extrinsic and intrinsic pathways are involved in TRAIL induced apoptosis. First, TRAIL binds to the death receptors TRAILR1 (DR4) and TRAILR2 (DR5), initiating the extrinsic pathway to activate caspase-8 and caspase-3, leading to cell death. Subsequently, the activated caspase-8 initiates the apoptotic intrinsic pathway.

IL-1β is involved in many kinds of cellular activities including migration, proliferation, and apoptosis^21,22^. Studies have shown that IL-1β could enhance the immunosuppressive properties of hBMMSCs in cell therapy^23^. In our previous research, we found that pre-activating hUCMSCs with IL-1β could induce membrane-bound TRAIL expression and enhance apoptosis of MDA-MB-231, MCF-7 and MDA-MB-453 breast cancer cell lines (unpublished data). In this study, we further investigated whether treatment of IL-1β on hUCMSCs could increase the expression of soluble TRAIL.

Cellular FADD-like IL-1β-converting enzyme inhibitory protein (cFLIP), a homolog of caspase-8, forms heterodimers with caspase-8 and blocks the activation of caspase-8 to regulate the death receptor-related extrinsic apoptosis pathway^24^. In humans, there are three isoforms of cFLIP: cFLIP_L_, cFLIP_S_ and cFLIP_R_. cFLIP_L_ is similar to pro-caspase-8 but lacks a functional caspase domain. cFLIP_S_ and cFLIP_R_ have two death-effector domains. Many studies had demonstrated that cFLIP is highly expressed in various human cancers, such as colorectal cancer, bladder urothelial cancer and breast cancer^25–27^, and the level of cFLIP expression was correlated with poor clinical outcomes. High expression of cFLIP_L_ has been found in most of the malignant cancer cells, and cFLIP_s_ was found in glioblastoma cells^28^. cFLIPR is specifically expressed in a number of cell lines and primary human T cells^29^. In addition, downregulation of FLIP enhances the FAS- or TRAIL-mediated cell apoptosis and the TRAIL sensitivity of cancers^27,30^. Given these findings, cFLIP is considered to be the master of the anti-apoptotic regulator of the FAS- or TRAIL-mediated apoptosis pathways and the resistant factor of cancers. Here we wanted to know the relationship between cFLIP and TRAIL resistance in breast cancer cell lines.

Embelin (2, 5-dihydroxy-3-undecyl-1, 4-benzoquinone) is a natural benzoquinone which is isolated from the *Embelia ribes*. Embelin has exhibited anti-inflammatory and anti-tumor effects in various cancer cells^31^. Embelin has also been found to be a cell-permeable, small-molecular weight inhibitor of X-linked inhibitor of apoptosis (XIAP), a target of apoptosis-resistance of cancer cells, and could hold great potential in cancer therapy^32,33^. The antitumor effect of embelin might manifest via inhibiting tumor growth, activating tumor suppressor gene p53 and inducing apoptosis^34–36^. Some studies have shown that embelin inhibited XIAP enhances TRAIL sensitivity in breast cancer cells and downregulates cFLIP_S_ to induce TRAIL-mediated cell apoptosis in malignant glioma cells^37,38^. We wondered if embelin could downregulate cFLIP to enhance the TRAIL sensitivity of breast cancer cells.

In this study, we investigated the relationship between the expression of cFLIP_L_ and TRAIL resistance in three different breast cancer cell lines. We further examined whether embelin could effectively downregulate the expression of cFLIP_L_. After co-culture with IL-1β-stimulated hUCMSCs, the effects of the secreted soluble TRAIL-mediated apoptosis in breast cancer cells was investigated. This study may provide a new strategy in breast cancer therapy.

## Results

### The impact of embelin on breast cancer cells

**Figure 1:**
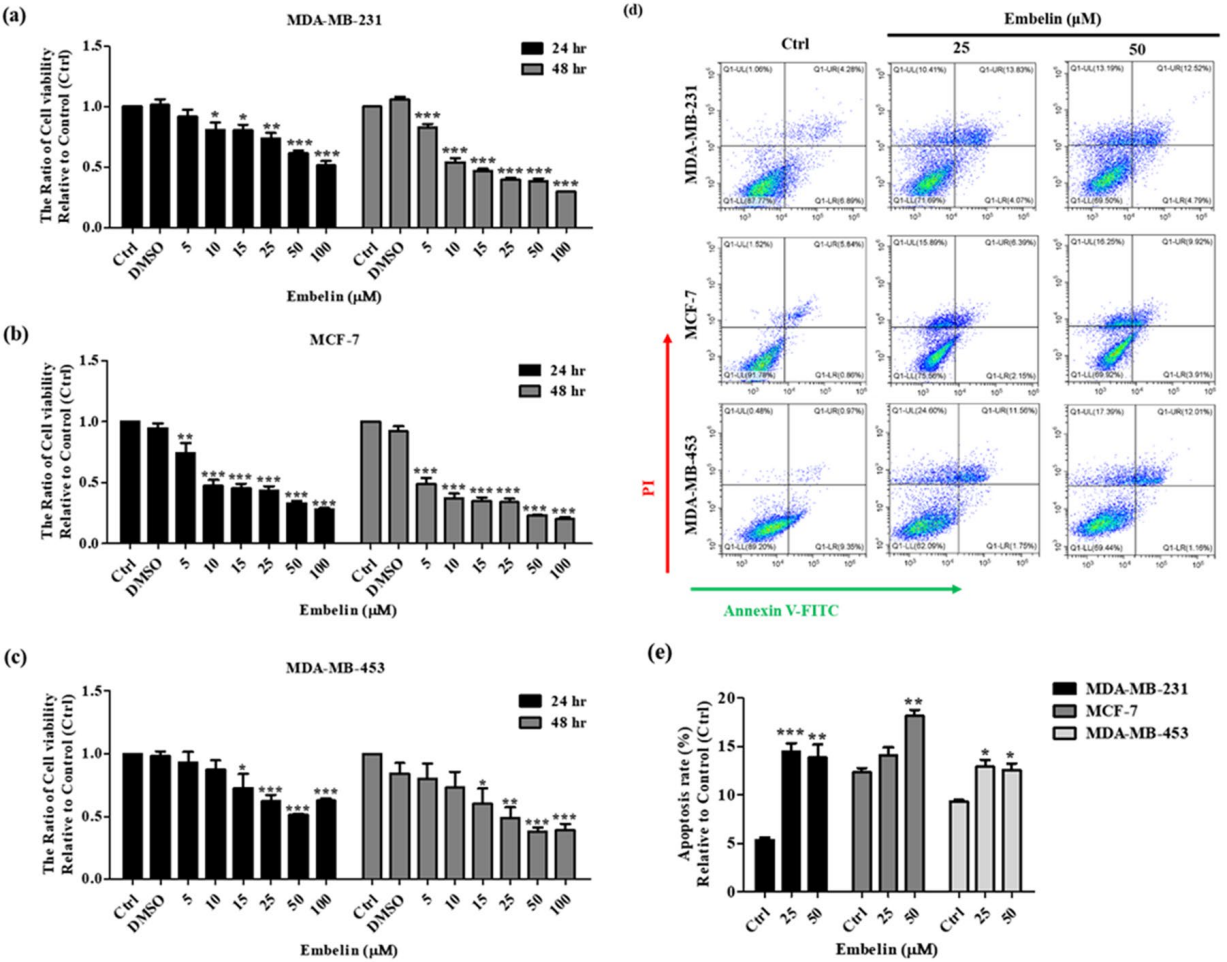
The effect of embelin on breast cancer cell lines. (**a**) MDA-MB-231, (**b**) MCF-7 and (**c**) MDA-MB-453 were treated with embelin at different concentrations (0, 5, 10, 15, 25, 50, 100 μM) for 24 h and 48 h. The results were detected by MTT assay and multimode microplate readers at a wavelength of 545 nm. (**d**) Annexin V/propidium (PI) dye was used to determine the rate of apoptosis following treatment with 25 or 50 μM embelin for 24 h in three breast cancer cell lines. (**e**) Quantitative graphs of the apoptotic rate of (**d**). Data were shown as mean ± SEM and analyzed by one-way ANOVA with Dunnett's test. (n = 3, *P < 0.05, **P < 0.01, ***P < 0.001).

We used an MTT assay to examine the cytotoxic impact of embelin within 0 to 100 μM concentration range for 24 and 48 h on three breast cancer cell lines MDA-MB-231, MCF-7, and MDA-MB-453. The results of treatment for 24 and 48 h showed that three breast cancer cell lines exhibited dose-dependent cytotoxicity in 0 to 50 μM treatment of embelin. After treatment with over 50 μM embelin for 24 h, the cytotoxicity of embelin on MDA-MB-453 showed no greater significant difference (Fig. 1a–c). There were significant changes in three breast cancer cell lines after treatment with 25 and 50 μM embelin for 24 h. Therefore, we focused on 25 and 50 μM treatment for 24 h to conduct further experiments. First, we investigated the effects of 25 and 50 μM embelin on breast cancer cells apoptosis by using flow cytometry. As shown in Fig. 1d, treatment of 25 and 50 μM embelin could induce the apoptosis of three breast cancer cell lines.

### Embelin downregulates cFLlP_L_ expression in breast cancer cells

**Figure 2:**
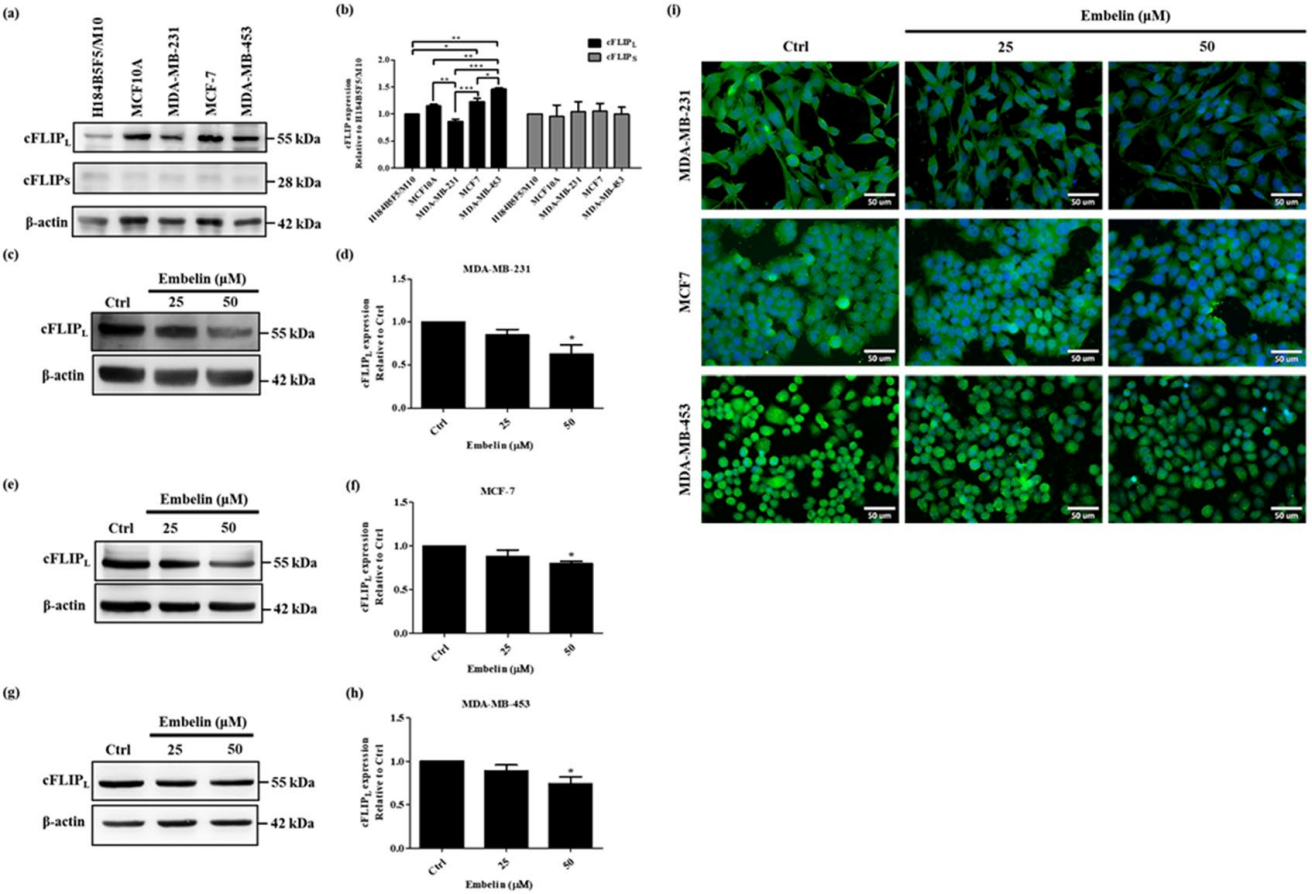
Embelin reduced cFLIP_L_ expression in breast cancer cells. (**a**) The protein expression of endogenous cFLIP_L_ and cFLIP_S_ in H184B5F5/M10, MCF10A, MDA-MB-231, MCF-7 and MDA-MB-453. (**b**) Quantitative graphs of the Western blot results of endogenous cFLIP protein expression of (**a**). Data were shown as mean ± SEM and analyzed by one-way ANOVA with Tukey's test. (n = 3, *P < 0.05, ***P < 0.001). The cFLIP_L_ expression of (**c**) MDA-MB-231, (**e**) MCF-7 and (**g**) MDA-MB-453 after treatment with 25 and 50 μM embelin for 24 h. (**d**, **f**, **h**) Quantitative graphs of the Western blot results of cFLIP_L_ expression of (**c**, **e**, **g**). (**i**) Immunocytochemistry staining for cFLIP_L_ in MDA-MB-231, MCF-7 and MDA-MB-453 with 25 and 50 μM embelin treatment for 24 h. The gray analysis of Western blots was normalized with β-actin. The full-length Western blots were shown in Supplementary Fig. S3. Data were shown as mean ± SEM and analyzed by one-way ANOVA with Dunnett's test. (n = 3, *P < 0.05, **P < 0.01, ***P < 0.001).

cFLIP is the anti-apoptotic regulator of the TRAIL-mediated apoptosis pathway and the resistant factor of cancers^39^. We wanted to know if embelin could downregulate cFLIP in breast cancer cells. Here we used two normal human mammary epithelial breast cell lines (H184B5F5/M10 and MCF10A) and three breast cancer cell lines to investigate the relationship between cFLIP_L_, cFLIP_S_, and TRAIL resistance in these cell lines. The previous study already found different TRAIL resistance in three breast cancer cell lines we used including TRAIL-sensitive MDA-MB-231, TRAIL-low resistant MCF-7, and TRAIL-high resistant MDA-MB-453^40^. The western blot data demonstrated that normal human breast epithelial cells expressed cFLIP_L_ and cFLIP_S._ cFLIP_L_ expression was increased as TRAIL-resistance increased and cFLIP_S_ was nonsignificant in three breast cancer cell lines (Fig. 2a,b). It also showed that cFLIP_L_ was correlated with the TRAIL resistance of three breast cancer cell lines. Then we treated breast cancer cells with 25 and 50 μM embelin for 24 h to investigate the effect of embelin on cFLIP_L_ expression. As shown in the western blot and immunofluorescence data, the expression of cFLIP_L_ exhibited a significant decrease in 50 μM embelin treatment of three breast cancer cell lines (Fig. 2c–i). Moreover, we detected the effect of embelin on two normal breast epithelial cell lines. The data showed that embelin did not downregulate the expression of cFLIP_L_ in H184B5F5/M10 and MCF10A cell lines (see Supplementary Fig. S1).

### Embelin increased caspase proteins activation in the intrinsic and extrinsic apoptotic pathway

**Figure 3:**
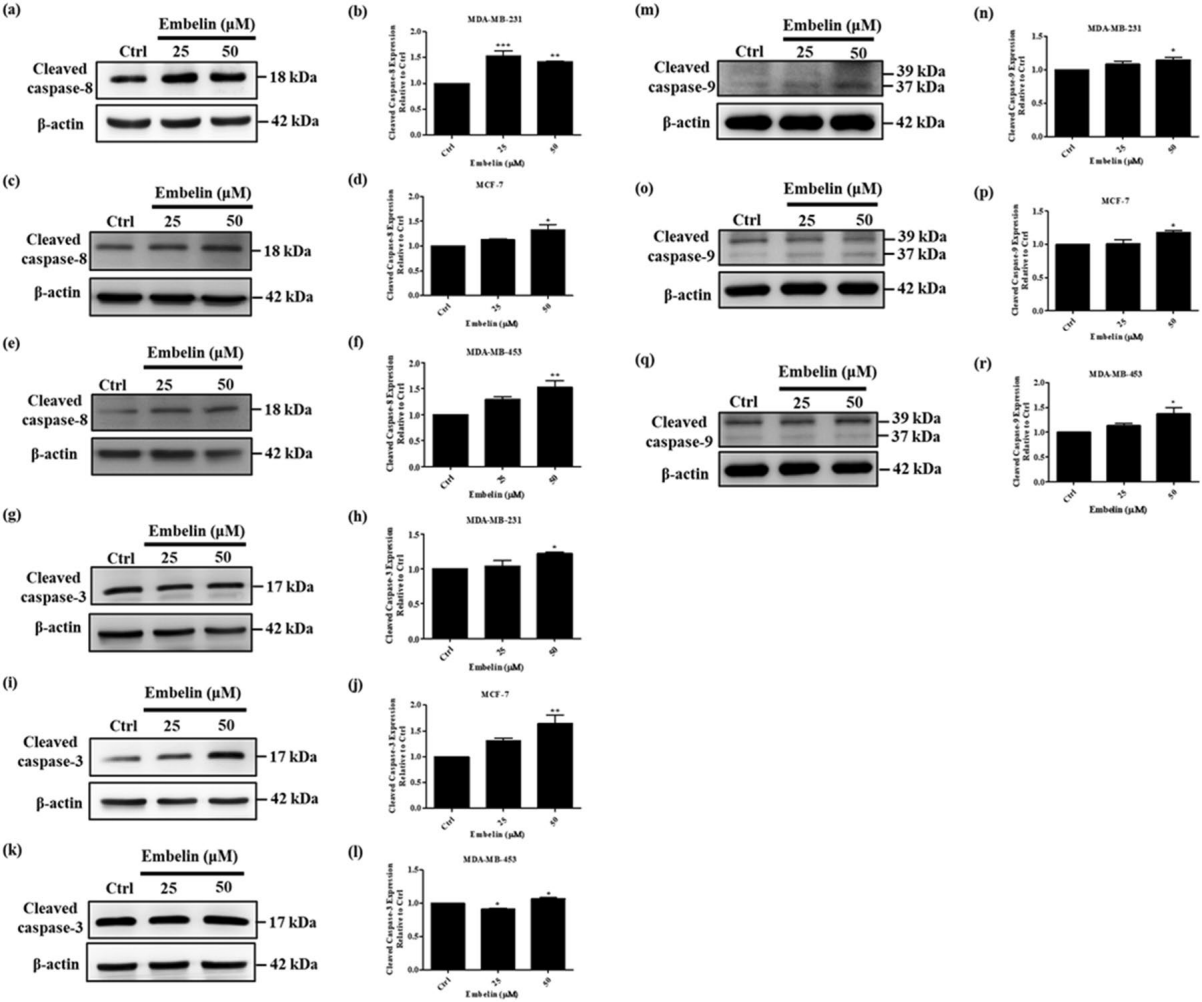
Embelin induced activation of caspase proteins in breast cancer cells. (**a**, **b**) MDA-MB-231 (**c**, **d**) MCF-7 and (**e**, **f**) MDA-MB-453 were treated with 25 and 50 μM embelin for 24 h. The cleaved caspase-8 expression and quantitative graphs of the Western blot results. (**g**, **h**) MDA-MB-231 (**i**, **j**) MCF-7 and (**k**, **l**) MDA-MB-453 were treated with 25 and 50 μM embelin for 24 h. The cleaved caspase-3 expression and quantitative graphs of the Western blot results. (**m**, **n**) MDA-MB-231 (**o**, **p**) MCF-7 and (**q**, **r**) MDA-MB-453 were treated with 25 and 50 μM embelin for 24 h. The cleaved caspase-9 expression and quantitative graphs of the Western blot results. The gray analysis of Western blots was normalized with β-actin. The full-length Western blots were shown in Supplementary Fig. S3. Data were shown as mean ± SEM and analyzed by one-way ANOVA with Dunnett's test. (n = 3, *P < 0.05, **P < 0.01, ***P < 0.001).

Previous studies have shown that cFLIP is involved in the extrinsic apoptotic pathway and that embelin could induce apoptosis of breast cancer cells^41,42^. To examine whether embelin could induce caspase proteins activation in the extrinsic apoptotic pathway on breast cancer cells, we treated MDA-MB-231, MCF-7, and MDA-MB-453 with 25 and 50 μM embelin for 24 h. The extrinsic apoptotic initiator caspase-8 and effector caspase-3 protein expressions in breast cancer cells were detected using Western blot. Cleaved caspase-8, the activated form of caspase-8, significantly increased in 50 μM embelin treatment of three breast cancer cell lines and increased only in 25 μM embelin-treated MDA-MB-231 (Fig. 3a–f). Cleaved caspase-3, the activated form of caspase-3, had a significant increase in three breast cancer cell lines at 50 μM embelin treatment and no significant difference at 25 μM treatment (Fig. 3g–l). We also investigated the intrinsic apoptotic initiator caspase-9 and cleaved caspase-9, the activated form of caspase-9. Cleaved caspase-9 significantly increased in 50 μM embelin treatment of three breast cancer cell lines. Therefore, we conclude thar embelin could induce the apoptosis of breast cancer cells through the intrinsic and extrinsic apoptotic pathways (Fig. 3m–r).

### IL-1β induces soluble TRAIL expression in hUCMSCs

**Figure 4:**
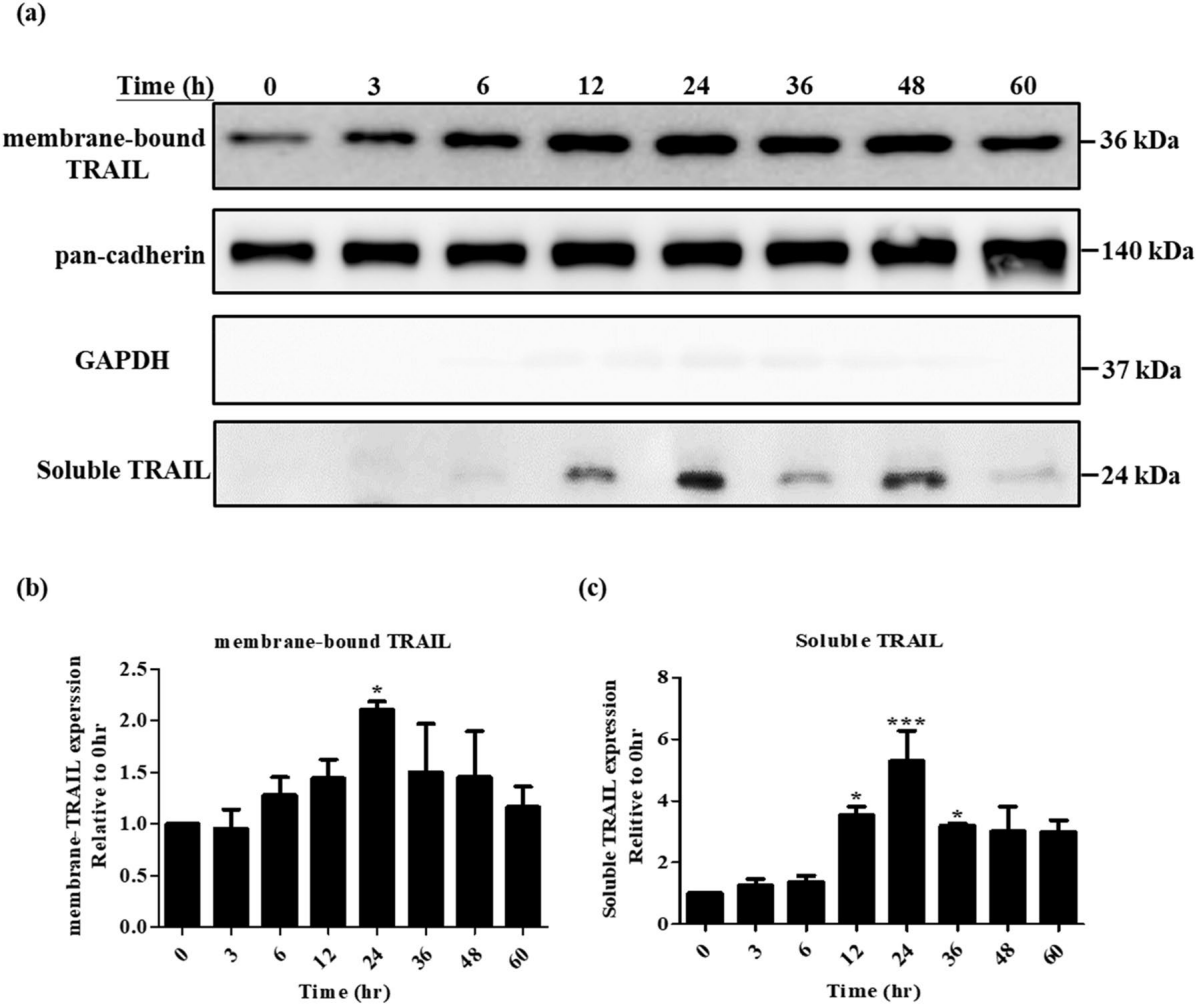
IL-1β induced the membrane-bound TRAIL protein and soluble TRAIL expression in hUCMSCs. (**a**) Time course of the membrane-bound TRAIL protein expression in hUCMSCs with 100 ng/ml IL-1β treatment analyzed by Western blot. (**b**) Quantitative graphs of membrane-bound TRAIL protein expression of (**a**). (**c**) Quantitative graphs of soluble TRAIL protein expression of (a). The gray analysis of Western blots of membrane-bound TRAIL was normalized with pan-cadherin. The full-length Western blots were shown in Supplementary Fig. S3. Data were shown as mean ± SEM and analyzed by one-way ANOVA with Dunnett's test. (n = 3, *P < 0.05, ***P < 0.001).

In our previous studies, the TRAIL expression of hUCMSCs can be highly induced by treatment with IL-1β (100 ng/ml) (unpublished data). First, we investigated the effect of IL-1β on hUCMSCs cytotoxicity and apoptosis by using MTT assay and flow cytometry. hUCMSCs were treated with 100 ng/ml IL-1β for 24 h. The result showed that IL-1β treatment had no significant effect on cell viability and apoptosis of hUCMSCs (see Supplementary Fig. S2). Furthermore, there was a finding that soluble TRAIL had higher apoptosis-inducing activity than membrane-bound TRAIL in cancer therapy^43^. Here we examined whether hUCMSCs could secrete soluble TRAIL and also be enhanced by treatment with 100 ng/ml IL-1β. hUCMSCs were treated with 100 ng/ml IL-1β for different durations (0, 3, 6, 12, 24, 36, 48, 60 h) and then the expression of soluble and membrane-bound TRAIL was detected by western blot. As the data show, soluble TRAIL emerged at 6 h and was significantly increased at 24 h (Fig. 4a,c). The membrane-bound TRAIL was continuously expressed and significantly increased at 12 h (Fig. 4a,b).

### Embelin-treated breast cancer cells and IL-1β-stimulated hUCMSCs enhance apoptosis

**Figure 5:**
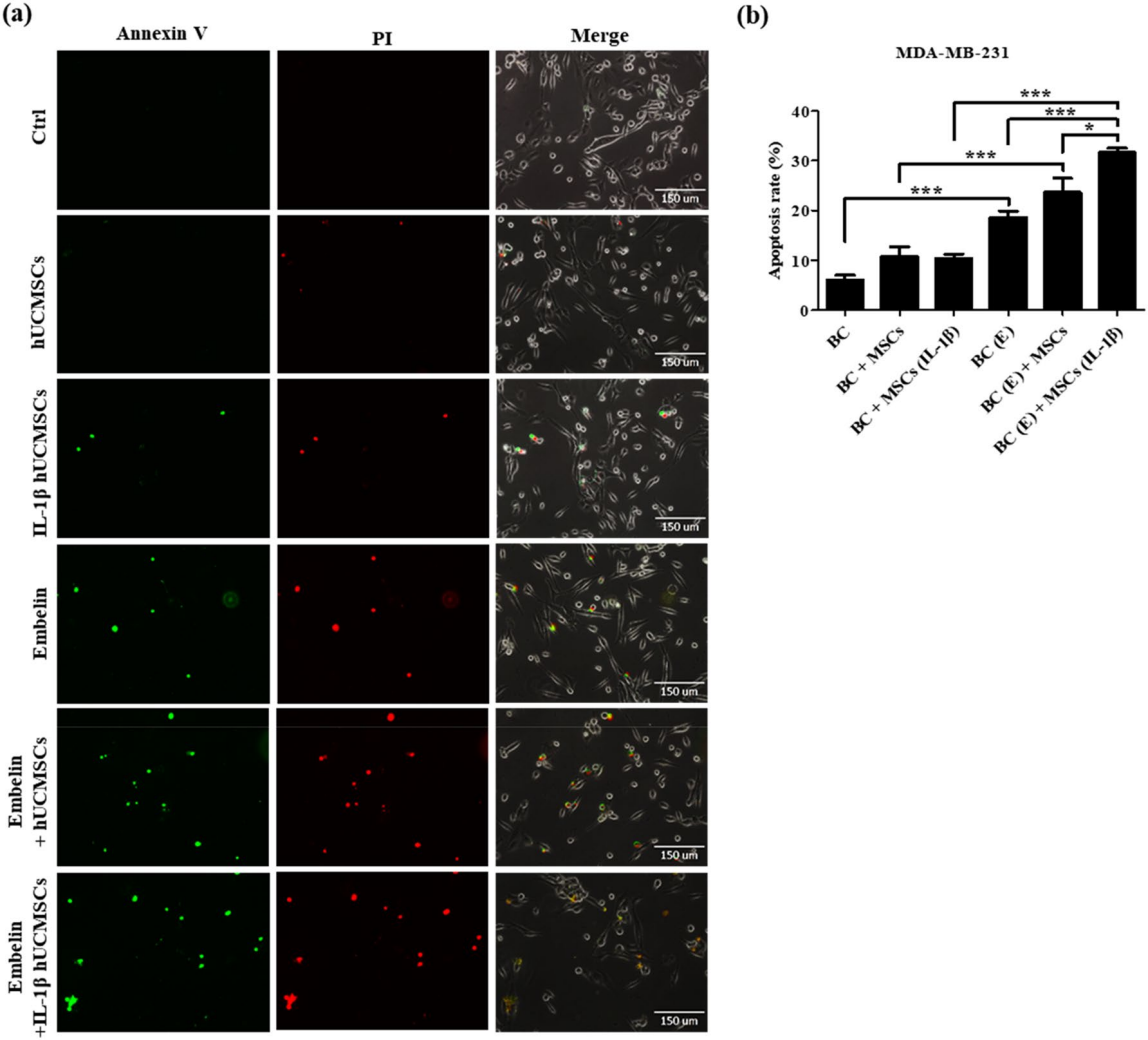

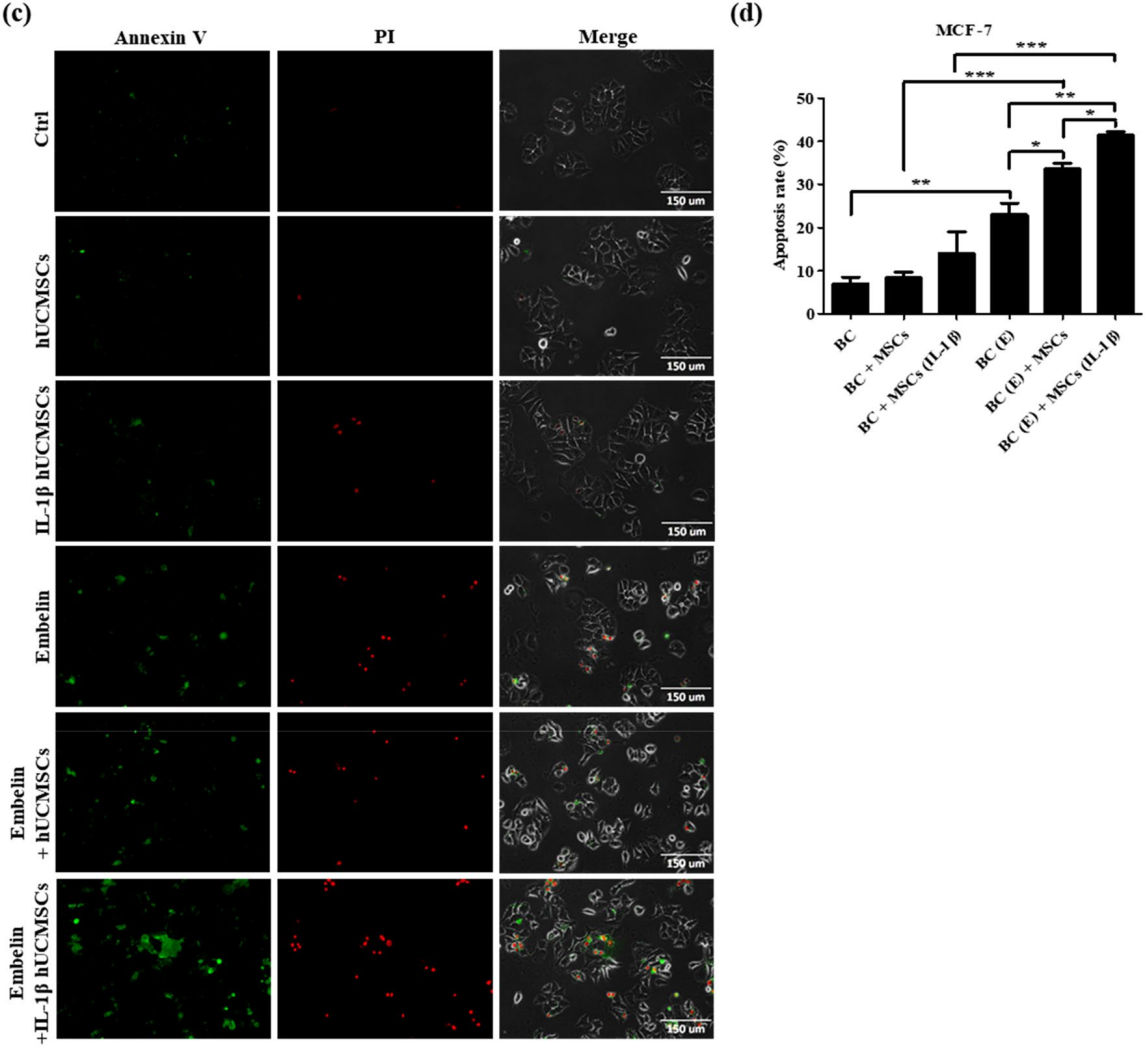

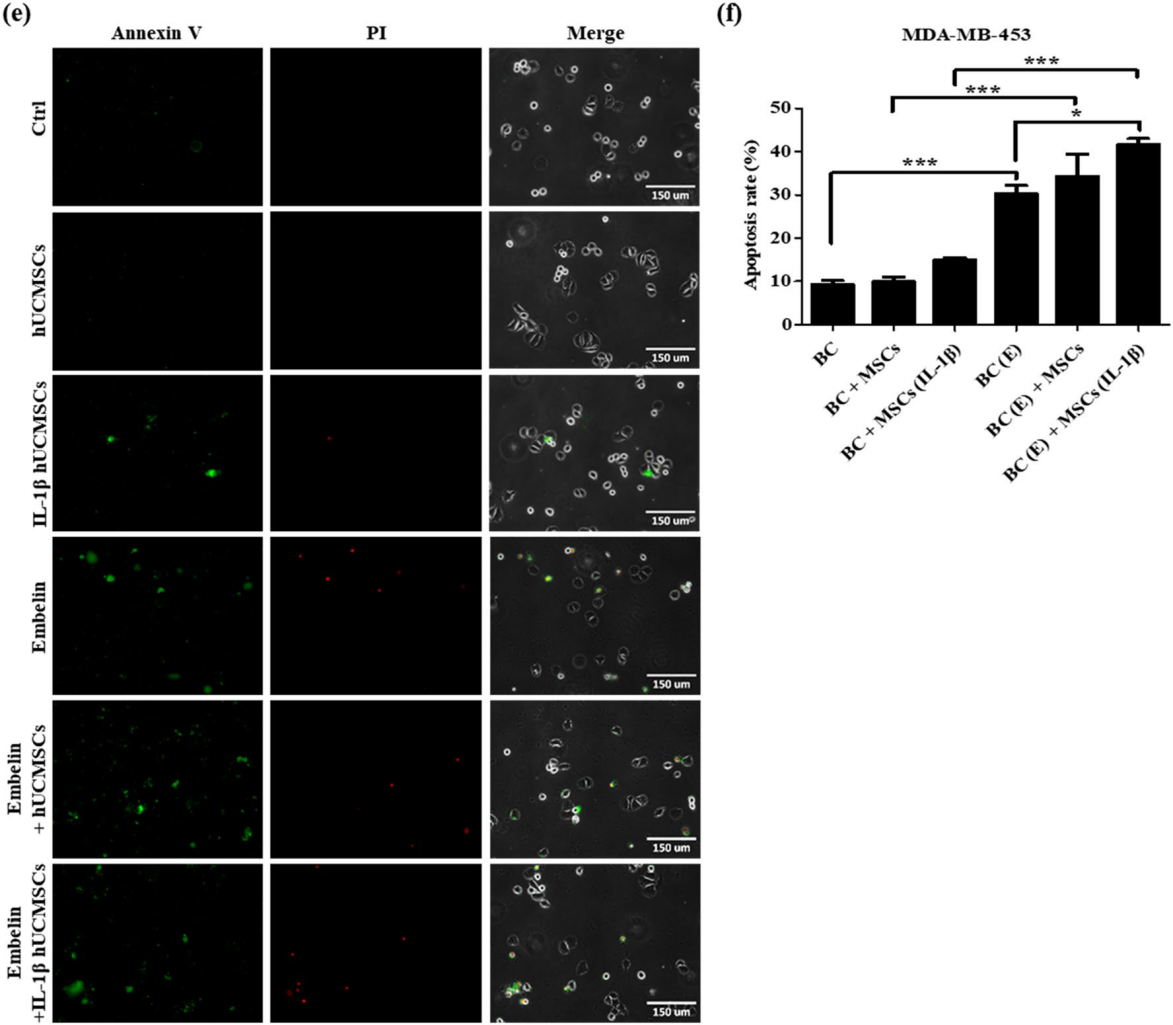
Embelin-treated breast cancer cells combined with IL-1β-stimulated hUCMSCs enhances the apoptosis of breast cancer cells. (**a**) MDA-MB-231, (**c**) MCF-7 and (**e**) MDA-MB-453 were pre-treated with or without 50 μM embelin and hUCMSCs were pretreated with or without 100 ng/ml IL-1β for 24 h. After co-culturing for 24 h, the expression levels of Annexin V (AV, green) and Propidium Iodide (PI, red) were detected by fluorescence microscopy. (**b**, **d**, **f**) The percentage of apoptotic cells was calculated by counting the AV+/PI−, AV+/PI+ expressing cells and total cell numbers. Data were shown as mean ± SEM and analyzed by one-way ANOVA with Dunnett's test. (n = 3, *P < 0.05, **P < 0.01, ***P < 0.001) (BC = untreated breast cancer cells, MSCs = hUCMSCs, BC (E) = breast cancer cells pretreated with 50 μM embelin, MSCs (IL-1β) = hUCMSCs pretreated with 100 ng/ml IL-1β).

According to our results, three breast cancer cell lines treated with 50 μM embelin for 24 h downregulated cFLIP_L_ expression (Fig. 2c–i) and increased caspase proteins activation (Fig. 3). hUCMSCs with 100 ng/ml IL-1β treatment for 24 h highly expressed soluble TRAIL (Fig. 4a). The transwell co-culture system was used to confirm the apoptosis rate of embelin**-**treated breast cancer cells and IL-1β-stimulated hUCMSCs. The apoptosis of breast cancer cells were detected using Annexin V-FITC and PI staining. Positive results for Annexin V-FITC meant early apoptotic cells and positive results for both Annexin V-FITC and PI meant late apoptotic cells. The results showed that embelin-treated TRAIL-sensitive MDA-MB-231 (Fig. 5a) and TRAIL-low resistant MCF-7 (Fig. 5c) had more late apoptotic cells. Embelin-treated TRAIL-high resistant MDA-MB-453 (Fig. 5e) had more early apoptotic cells. In three breast cancer cell lines, the apoptotic rate was similar in breast cancer cells only, breast cancer cells co-cultured with hUCMSCs and breast cancer cells co-cultured with IL-1β-stimulated hUCMSCs groups (Fig. 5b,d,f). Co-culturing embelin-treated breast cancer cells with hUCMSCs or with IL-1β-stimulated hUCMSCs led to more apoptotic cells than co-culturing untreated breast cancer cells with hUCMSCs or with IL-1β-stimulated hUCMSCs in all three cell lines (Fig. 5b,d,f). Elevated apoptotic cell population was found in embelin-treated breast cancer cells co-cultured with IL-1β-stimulated hUCMSCs and the apoptotic rate was nearly 30% in MDA-MB-231 cell line, and 40% in MCF-7 and MDA-MB-453 cell lines (Fig. 5b,d,f). Furthermore, we calculated the combination index (CI)^44^ to determine the extent to which embelin with IL-1β-stimulated hUCMSCs (BC(E) + MSCs (IL-1β) enhance apoptotic activity of breast cancer cells. The numerical value of CI calculated with apoptotic rate in Fig. 5, CI (MDA-MB-231) = [(18 − 6) + (11 − 6)]/(32 − 6) = 0.65; CI (MCF-7) = [(23 − 7) + (14 − 7)]/(42 − 7) = 0.66; CI (MDA-MB-453) = [(31 − 9) + (15 − 9)]/(42 − 9) = 0.85. In these three breast cancer cell lines, the numerical value of CI was less than one, which indicates synergism in the combinations of embelin and IL-1β-stimulated hUCMSCs in increasing breast cancer cell apoptosis (CI < 1). These results demonstrate that embelin enhanced TRAIL sensitivity and cell apoptosis in both TRAIL-sensitive and TRAIL-resistant breast cancer cell lines. The soluble TRAIL secreted from IL-1β-stimulated hUCMSCs effectively increased cell apoptosis in embelin-treated breast cancer cell lines.

## Discussion

TRAIL is a potent inducer of apoptosis in various cancer cells but not in normal cells. Due to its ability to selectively induce cancer cell death, there is growing interest in TRAIL for use in cancer therapy^43,45^. TRAIL-mediated apoptosis initiates at the extrinsic apoptosis pathway by binding to death receptors DR4 and DR5^46^. It has been found that embelin, the XIAP inhibitor, induces TRAIL-mediated apoptosis by upregulating DR4 and DR5 expression in human leukemia cells^47^. In our previous studies, we found that embelin could upregulate DR4 and DR5 expression and enhance TRAIL-mediated apoptosis in breast cancer cell lines (unpublished data).

The role of cFLIP_L_ in apoptosis is connected with the death receptor-related extrinsic apoptosis pathway. Accumulating reports showed that cFLIP is correlated with TRAIL-resistance and a regulator of TRAIL-mediated apoptosis. We show that the expression level of cFLIP_L_ is related to TRAIL-resistance in three different TRAIL-resistant breast cancer cell lines (Fig. 2a,b). The expression of cFLIP_S_ is unrelated to the TRAIL resistance in these cell lines. Embelin has been reported to enhance TRAIL-mediated apoptosis not only by upregulating the death receptors DR4 and DR5 in breast cancer cells, but also by downregulating cFLIP_S_ in malignant glioma cells. In this study, we found that embelin significantly downregulated cFLIP_L_ in breast cancer cell lines (Fig. 2c–i). In addition, embelin caused dose-dependent death of breast cancer cells and upregulated cleaved caspase-8, cleaved caspase-9 and cleaved caspase-3 expression (Figs. 1, 3).

Because of the antitumor effect and the homing ability of MSCs, stem cell therapy is considered to have great potential for cancer therapy. Various antitumor effects of hUCMSCs have been observed such as inhibition of proliferation, modulation of the cell cycle, and inducement of apoptosis through the secretion of related proteins. Research has found that the conditioned medium of hUCMSCs could induce cell apoptosis in human lung cancer cell lines, human hepatocellular cancer cell lines^12^, and human glioma cell lines^48^. In our previous studies, we had found that naïve hUCMSCs expressed membrane-bound TRAIL; the expression of membrane-bound TRAIL was upregulated after being treated with IL-1β (unpublished data). Here, we have also provided evidence that hUCMSCs expressed membrane-bound TRAIL and soluble TRAIL, and that both can be enhanced by IL-1β treatment (Fig. 4). The function of soluble TRAIL secreted from hUCMSCs in embelin treated breast cancer cells was further investigated by co-culturing these cells. In three breast cancer cell lines, the group of co-cultured embelin-treated breast cancer cells and IL-1β induced hUCMSCs, enhanced nearly 40% of apoptotic cells including early apoptosis and late apoptosis. These data suggested that embelin downregulating cFLIP_L_ could enhance TRAIL sensitivity of breast cancer cells and the soluble TRAIL secretion by hUCMSCs could effectively increase the apoptosis of breast cancer cells.

In summary, our data demonstrated that the cFLIP_L_ is correlated with TRAIL resistance in breast cancer cells. Embelin downregulated cFLIP_L_ to enhance the TRAIL sensitivity of breast cancer cells and induced the caspase proteins activation in the extrinsic and intrinsic apoptotic pathways. In addition, hUCMSCs expressed the membrane form and soluble form of TRAIL protein induced by IL-1β treatment. Co-cultured embelin-treated breast cancer cells and IL-1β-stimulated hUCMSCs exert a synergistic effect in increasing TRAIL-mediated apoptosis. Above all, this research presents an effective way of utilizing embelin and IL-1β-stimulated hUCMSCs in cancer therapy of different TRAIL-resistant breast cancer cells.

## Methods

### Breast cancer cell lines and cell culture

Human breast cancer cells MDA-MB-231, MCF-7 and MDA-MB-453 were obtained from Bioresource Collection and Research Center (BCRB), Hsinchu, Taiwan. Three breast cancer cell lines were cultured in Dulbecco's Modified Eagle Medium: Nutrient Mixture F-12 (DMEM/F12; Life technology, NY, USA) with 10% fetal bovine serum (Thermo, Logan, UT), 1% penicillin–streptomycin solution (Corning, NY, USA) and incubated at 5% CO_2_ and 37 °C. When cells reached 80% confluence, the cells were detached using HyClone™ HyQTase Cell Detachment Reagent (Thermo, Logan, UT) and replated 8 × ${10}^{5}$ cells per 100 mm petri dish.

### Human umbilical cord mesenchymal stem cells (hUCMSCs)

Human umbilical cord mesenchymal stem cells were purchased from the BCRC. hUCMSCs were cultured in 56% Dulbecco's Modified Eagle Medium (DMEM-LG; Life technology, NY, USA), 37% MCBD201 (Sigma, MO, USA), 2% fetal bovine serum (Thermo, Logan, UT), 1x Insulin–Transferrin–Selenium-A (Life technology, NY, USA), 1x antibiotic–antimycotic solution (Corning, NY, USA), 10 nM Dexamethasone (Sigma, MO, USA), 0.5 mg/ml of Bovine Serum Albumin Fraction V (Sigma, MO, USA), 50 μM l-ascorbic acid 2-phosphate sesquimagnesium salt hydrate (Sigma, MO, USA), 10 ng/ml Epidermal growth factor (PeproTech, NJ, USA), 1 ng/ml of platelet-derived growth factor-BB (PeproTech, NJ, USA), and sterilized water to total volume. The cells were detached by using HyClone™ HyQTase Cell Detachment Reagent (Thermo, Logan, UT). When cells reached 80% confluence, they were replated 8 × ${10}^{5}$ cells per 100 mm petri dish.

### MTT cell viability assay

Human breast cancer cells MDA-MB-231, MCF-7 and MDA-MB-453 were seeded 3 × ${10}^{4}$ cells per well in 96-well plates with culture medium for 24 h and then starved in serum-free DMEM-F12 for 16 h. hUCMSCs were seed 1 × 10^4^ per well in 96-well plated with cultured medium for 24 h, then starved in DMED-LG with 1% FBS for 16 h. After starvation, breast cancer cells were treated with embelin at different concentrations (0–100 μM) for 24 and 48 h. hUCMSCs were treated with 100 ng/ ml IL-1β for 24 h. Then cells were incubated with 1 mg/ml MTT (3-(4,5-Dimethylthiazol-2-yl)-2,5-diphenyltetrazolium bromide; USB, Cleveland, OH, USA) reagent for 4 h at 5% CO_2_ and 37 °C. The reagent was removed and the crystal was dissolved with dimethyl sulfoxide (DMSO; Bio Basic, YTO, CA) on the shaker for 30 min at 37 °C. The results were detected with Multimode microplate readers Infinite 200 (TECAN, Switzerland).

### Treatment of cancer cells and MSCs

Human breast cancer cells MDA-MB-231, MCF-7 and MDA-MB-453 were plated 1 × ${10}^{6}$ cells in 60 mm petri dish with culture medium for 24 h and then starved in serum-free DMEM-F12 for 16 h. After starvation, Cells treated with 0, 25 and 50 μM embelin for 24 h. hUCMSCs were seeded 8 × ${10}^{5}$ cells in 60 mm petri dishes with culture medium for 24 h and then starved in 1% fetal bovine serum DMEM-LG for 16 h. Afterward, cells were treated with 100 ng/ml human recombinant Interleukin-1β (IL-1β; PeproTech, NJ, USA) for different durations.

### Western blotting

Cells were washed with PBS twice and lysed using M-PER Mammalian Protein Extraction Reagent (Thermo, IL, USA) with 1% Halt Protease Inhibitor Cocktail (Thermo, IL, USA). The membrane proteins were fractionated by Mem-PER™ Plus Membrane Protein Extraction Kit (Thermo, IL, USA), according to the manufacturer’s instructions. The extraction was vortexed for 5 min and centrifuged at 14,000 g for 15 min at 4 °C. Protein concentrations were determined by Bio-Rad Protein Assay Dye Reagent (BIO-RAD, CA, USA) and multimode microplate readers (Infinite 200, TECAN). To collect soluble TRAIL, hUCMSCs were cultured in 60 mm petri dishes with/without IL-1β treatment for 0–60 h in 2 ml of media. Proteins were concentrated by using Amicon® Ultra-4 centrifugal filter unit (Merck Millipore, NJ, USA). Protein samples (25 μg) or equal amount concentrated medium (25 μl) were separated by 10% or 15% sodium dodecyl sulfate–polyacrylamide gel electrophoresis (SDS-PAGE) and transferred to Immun-Blot PVDF Membranes (BIO-RAD, CA, USA). After transfer, membranes were blocked with 5% Fish Gelatin Blocking Buffer (AMRESCO, OH, USA) in Tris-buffered saline with tween 20 (TBST) for an hour at room temperature (RT). Then membranes were washed with TBST for 10 min three times and incubated with cFLIP antibody (Abcam, Cambridge, UK) diluted at 1:3000, caspase-8 antibody (GeneTex, CA, USA) diluted at 1:3000, caspase-3 antibody (GeneTex, CA, USA) diluted at 1:3000, caspase-9 antibody (Cell Signaling, MA, USA) diluted at 1:1000, TRAIL antibody (Cell Signaling, MA, USA) diluted at 1:2500, pan-cadherin antibody (Abcam, Cambridge, UK) diluted at 1:4000, GAPDH antibody (Cell Signaling, MA, USA) diluted at 1:4000 and beta-actin antibody (GeneTex, CA, USA) diluted at 1:10,000 in blocking buffer at 4 °C overnight. The membranes were washed with TBST for 10 min three times and incubated with Rabbit IgG antibody (HRP) (GeneTex, CA, USA) for an hour at RT. The results were detected by enhanced chemiluminescence substrate (ECL) (T-Pro Biotechnology, New Taipei County, Taiwan) and LAS-4000 Luminescence Imaging System (GE, CT, USA). The blots were analyzed using AlphaEaseFC 4.0 software and the full-length Western blots were shown in Supplementary Fig. S3.

### Cell immunofluorescence and images

Human breast cancer cells MDA-MB-231, MCF-7 and MDA-MB-453 were seeded 1 × ${10}^{5}$ cells on 12 mm glass coverslips in 24 well plates with culture medium for 24 h and then starved in serum-free DMEM-F12 for 16 h. After starvation, cells were treated with 0, 25, 50 μM embelin for 24 h. Cells were washed for 5 min three times and fixed with 4% paraformaldehyde (Ferak, Berlin, Germany) in PBS for 30 min at RT, then washed in 4% paraformaldehyde with PBS for 5 min three times. Cells were permeabilized with 0.1% Triton X-100 (Sigma, MO, USA) for 15 min at RT. Cells were washed with PBS for 5 min three times and blocked with 2% bovine serum albumin (BSA; Sigma, MO, USA) in PBS for 30 min at RT. After blocking, cells were incubated with primary antibody cFLIP antibody (Abcam, Cambridge, UK) diluted at 1:250 in the blocking buffer at 4 °C overnight. Next, cells were washed with PBS for 5 min three times and incubated with secondary antibody Alexa Fluor 488-AffiniPure Goat Anti-Rabbit IgG (Jackson ImmunoResearch Labs, West Grove, PA, USA) diluted at 1:500 for an hour at RT. Cells were stained with Hoechst 33,258 (Sigma, MO, USA) diluted at 1:5000 for 5 min at RT after washing with PBS 5 min for three times. Finally, cells were washed with PBS for 5 min three times and mounted by Fluorescence Mounting Medium (Ibidi, Planegg, Germany). The images were captured by using a Fluorescent Microscope (Leica DM6000B, Wentzler, Germany).

### Flow cytometry

Breast cancer cells were seed 3 × 10^5^ cells in 6 well plates with culture medium for 24 h and then starved in serum-free DMEM-F12 for 16 h. hUCMSCs were seeded 2.5 × 10^5^ cells in 6 cm dishes culture medium for 24 h and then starved in DMEM-LG medium with 1% FBS for 16 h. After starvation, breast cancer cells were treated with 25, 50 μM embelin and hUCMSCs were treated with 100 ng/ml IL-1β for 24 h. The cells were washed twice with PBS and detached by HyQtase (Thermo, Logan, UT). Cells were collected and stained with 2% Annexin V-FITC (AV) and 2% Propidium Iodide (PI) in 100 μl of binding buffer for 10 min in the dark. 400 μl of binding buffer was added and transferred to the flow tubes. The results were analyzed by using Beckman Coulter CytoFLEX (Beckman, IL, USA).

### Transwell co-culture system and apoptosis detection

Human breast cancer cells were seeded 2 × ${10}^{5}$ cells on 16 mm glass coverslips in 6 well plates with the culture medium for 24 h and then starved in serum-free DMEM-F12 for 16 h. hUCMSCs were seeded 1 × ${10}^{5}$ cells in 0.4 μm pore, 24 mm transwell inserts with the culture medium for 24 h and starved for 16 h in DMEM-LG medium with 1% FBS. Then breast cancer cells were pretreated with 50 μM embelin and hUCMSCs were pretreated with100 ng/ml IL-1β for 24 h. After pretreatment, the transwell inserts were moved into 6 well plates to co-culture breast cancer cells and hUCMSCs for 24 h in DMEM-LG with 1% FBS. The coverslips were removed and Annexin V-FITC Apoptosis Detection Kit (Strong Biotech Corporation, TPE, ROC) were used to detect apoptosis. Breast cancer cells were stained with 2% Annexin V-FITC (AV) and 2% Propidium Iodide (PI) in a binding buffer for 10 min in the dark. After staining, the images were immediately captured by using a Fluorescent Microscope (Leica DM6000B, Wentzler, Germany). The apoptotic rate in Fig. 5 was determined by calculating the proportion of cells that have undergone apoptosis (cell numbers of Annexin V+/PI−, Annexin V+/PI+ expressing cells) in relation to the total number of cells in five randomly selected fields of three independent experiments.

### Statistical analysis

All data were represented as mean ± SEM from three independent experiments. Statistical analysis was analyzed by one-way ANOVA using Prism 5 software. *P* values < 0.05 were considered statistically significant by Dunnett's test. The combination index (CI) was calculated with the apoptotic rate using following equation:

$$CI=\frac{ \left[\mathrm{BC}\left(\mathrm{E}\right)-\mathrm{BC}\right]+\left\{\left[\mathrm{ BC}+\mathrm{ MSCs }\left(\mathrm{IL}-1\upbeta \right)\right]-\mathrm{BC}\right\}}{\left\{\left[\mathrm{BC}\left(\mathrm{E}\right)+\mathrm{MSCs }\left(\mathrm{IL}-1\upbeta \right)\right]-\mathrm{BC}\right\}}$$

## Supplementary Information

Supplementary Information.

## Data Availability

The data used to support the findings of this study are included within the article.

## References

[1] Malvezzi M (2019). European cancer mortality predictions for the year 2019 with focus on breast cancer. Ann. Oncol..

[2] Alfano CM (2017). Inflammatory cytokines and comorbidity development in breast cancer survivors versus noncancer controls: Evidence for accelerated aging?. J. Clin. Oncol..

[3] Kalimutho M (2015). Targeted therapies for triple-negative breast cancer: Combating a stubborn disease. Trends Pharmacol. Sci..

[4] Bush TL, Whiteman M, Flaws JA (2001). Hormone replacement therapy and breast cancer: A qualitative review. Obstet. Gynecol..

[5] Kim K, Fisher MJ, Xu S-Q, El-Deiry WS (2000). Molecular determinants of response to TRAIL in killing of normal and cancer cells. Clin. Cancer Res..

[6] Rahman M (2009). TRAIL induces apoptosis in triple-negative breast cancer cells with a mesenchymal phenotype. Breast Cancer Res. Treat..

[7] Bianco P, Robey PG, Simmons PJ (2008). Mesenchymal stem cells: Revisiting history, concepts, and assays. Cell Stem Cell.

[8] Rastegar F (2010). Mesenchymal stem cells: Molecular characteristics and clinical applications. World J. Stem Cells.

[9] Subramanian A (2012). Human umbilical cord Wharton's jelly mesenchymal stem cells do not transform to tumor-associated fibroblasts in the presence of breast and ovarian cancer cells unlike bone marrow mesenchymal stem cells. J. Cell. Biochem..

[10] Ayuzawa R (2009). Naive human umbilical cord matrix derived stem cells significantly attenuate growth of human breast cancer cells in vitro and in vivo. Cancer Lett..

[11] Ding D-C, Chang Y-H, Shyu W-C, Lin S-Z (2015). Human umbilical cord mesenchymal stem cells: A new era for stem cell therapy. Cell Transplant..

[12] Yuan Y (2018). Suppression of tumor cell proliferation and migration by human umbilical cord mesenchymal stem cells: A possible role for apoptosis and Wnt signaling. Oncol. Lett..

[13] Wu S, Ju G-Q, Du T, Zhu Y-J, Liu G-H (2013). Microvesicles derived from human umbilical cord Wharton’s jelly mesenchymal stem cells attenuate bladder tumor cell growth in vitro and in vivo. PLoS ONE.

[14] Kim SM (2008). Gene therapy using TRAIL-secreting human umbilical cord blood-derived mesenchymal stem cells against intracranial glioma. Can. Res..

[15] Sartoris S (2010). Efficacy assessment of interferon-alpha—Engineered mesenchymal stromal cells in a mouse plasmacytoma model. Stem Cells Dev..

[16] Levy O (2013). mRNA-engineered mesenchymal stem cells for targeted delivery of interleukin-10 to sites of inflammation. Blood.

[17] Turner A (2013). MADD knock-down enhances doxorubicin and TRAIL induced apoptosis in breast cancer cells. PLoS ONE.

[18] Wiley SR (1995). Identification and characterization of a new member of the TNF family that induces apoptosis. Immunity.

[19] Lowe SW, Cepero E, Evan G (2004). Intrinsic tumour suppression. Nature.

[20] Kumar R, Herbert P, Warrens A (2005). An introduction to death receptors in apoptosis. Int. J. Surg..

[21] Joos H, Wildner A, Hogrefe C, Reichel H, Brenner RE (2013). Interleukin-1 beta and tumor necrosis factor alpha inhibit migration activity of chondrogenic progenitor cells from non-fibrillated osteoarthritic cartilage. Arthritis Res. Ther..

[22] Kuida K (1995). Altered cytokine export and apoptosis in mice deficient in interleukin-1 beta converting enzyme. Science.

[23] Opitz CA (2009). Toll-like receptor engagement enhances the immunosuppressive properties of human bone marrow-derived mesenchymal stem cells by inducing indoleamine-2, 3-dioxygenase-1 via interferon-β and protein kinase R. Stem Cells.

[24] Kataoka T (2005). The caspase-8 modulator c-FLIP. Crit. Rev.™ Immunol..

[25] Wilson TR (2007). c-FLIP: A key regulator of colorectal cancer cell death. Can. Res..

[26] Korkolopoulou P (2004). c-FLIP expression in bladder urothelial carcinomas: Its role in resistance to Fas-mediated apoptosis and clinicopathologic correlations. Urology.

[27] Day TW, Huang S, Safa AR (2008). c-FLIP knockdown induces ligand-independent DR5-, FADD-, caspase-8-, and caspase-9-dependent apoptosis in breast cancer cells. Biochem. Pharmacol..

[28] Bagnoli M, Canevari S, Mezzanzanica D (2010). Cellular FLICE-inhibitory protein (c-FLIP) signalling: A key regulator of receptor-mediated apoptosis in physiologic context and in cancer. Int. J. Biochem. Cell Biol..

[29] Golks A, Brenner D, Fritsch C, Krammer PH, Lavrik IN (2005). c-FLIPR, a new regulator of death receptor-induced apoptosis. J. Biol. Chem..

[30] Hyer ML (2002). Downregulation of c-FLIP sensitizes DU145 prostate cancer cells to Fas-mediated apoptosis. Cancer Biol. Ther..

[31] Chitra M, Sukumar E, Suja V, Devi S (1994). Antitumor, anti-inflammatory and analgesic property of embelin, a plant product. Chemotherapy.

[32] Nikolovska-Coleska Z (2004). Discovery of embelin as a cell-permeable, small-molecular weight inhibitor of XIAP through structure-based computational screening of a traditional herbal medicine three-dimensional structure database. J. Med. Chem..

[33] Chen J, Nikolovska-Coleska Z, Wang G, Qiu S, Wang S (2006). Design, synthesis, and characterization of new embelin derivatives as potent inhibitors of X-linked inhibitor of apoptosis protein. Bioorg. Med. Chem. Lett..

[34] Nigam N (2015). Targeting mortalin by embelin causes activation of tumor suppressor p53 and deactivation of metastatic signaling in human breast cancer cells. PLoS ONE.

[35] Coutelle O (2014). Embelin inhibits endothelial mitochondrial respiration and impairs neoangiogenesis during tumor growth and wound healing. EMBO Mol. Med..

[36] Park S-Y (2013). Embelin induces apoptosis in human glioma cells through inactivating NF-κB. J. Pharmacol. Sci..

[37] Allensworth JL, Aird KM, Aldrich AJ, Batinic-Haberle I, Devi GR (2012). XIAP inhibition and generation of reactive oxygen species enhances TRAIL sensitivity in inflammatory breast cancer cells. Mol. Cancer Ther..

[38] Siegelin M, Gaiser T, Siegelin Y (2009). The XIAP inhibitor embelin enhances TRAIL-mediated apoptosis in malignant glioma cells by down-regulation of the short isoform of FLIP. Neurochem. Int..

[39] Kim Y, Suh N, Sporn M, Reed JC (2002). An inducible pathway for degradation of FLIP protein sensitizes tumor cells to TRAIL-induced apoptosis. J. Biol. Chem..

[40] Keane MM, Ettenberg SA, Nau MM, Russell EK, Lipkowitz S (1999). Chemotherapy augments TRAIL-induced apoptosis in breast cell lines. Can. Res..

[41] Mori T (2007). Effect of the XIAP inhibitor embelin on TRAIL-induced apoptosis of pancreatic cancer cells. J. Surg. Res..

[42] Safa AR (2013). Roles of c-FLIP in apoptosis, necroptosis, and autophagy. J. Carcinog. Mutagen..

[43] Almasan A, Ashkenazi A (2003). Apo2L/TRAIL: Apoptosis signaling, biology, and potential for cancer therapy. Cytokine Growth Factor Rev..

[44] Chou T-C (2010). Drug combination studies and their synergy quantification using the Chou–Talalay method. Can. Res..

[45] Kruyt FA (2008). TRAIL and cancer therapy. Cancer Lett..

[46] Pan G (1997). The receptor for the cytotoxic ligand TRAIL. Science.

[47] Hu R (2015). The XIAP inhibitor embelin enhances TRAIL-induced apoptosis in human leukemia cells by DR4 and DR5 upregulation. Tumor Biol..

[48] Yang C (2014). Conditioned media from human adipose tissue-derived mesenchymal stem cells and umbilical cord-derived mesenchymal stem cells efficiently induced the apoptosis and differentiation in human glioma cell lines in vitro. BioMed Res. Int..

