# Supplementary material for: Embelin downregulated cFLIP in breast cancer cell lines facilitate anti-tumor effect of IL-1β-stimulated human umbilical cord mesenchymal stem cells
Source: Sci Rep. 2021 Jul 19;11:14720. doi: 10.1038/s41598-021-94006-w (PMC8289868; doi:10.1038/s41598-021-94006-w)

Embelin Downregulated cFLIP in Breast Cancer Cell Lines Facilitate Anti-tumor Effect of IL-1β-stimulated Human Umbilical Cord Mesenchymal Stem Cells

Ya-Han Liang[1*], Jiann-Ming Wu[2*], Jui-Wen Teng[1], Eric Hung[1], Hwai-Shi Wang[1]

Supplementary Figure 1. Effect of Embelin on the expression of $cFLIP_L$ expression in normal breast epithelial cell lines.

The $cFLIP_L$ expression of (a) H184B5F5/M10 and (c) MCF10A after treatment with 25 and 50 μM embelin for 24 hours. (b, d) Quantitative graphs of the Western blot results of $cFLIP_L$ expression of (a, c) The gray analysis of Western blots was normalized with β-actin. The full-length Western blots were shown in Supplementary Fig. S3.

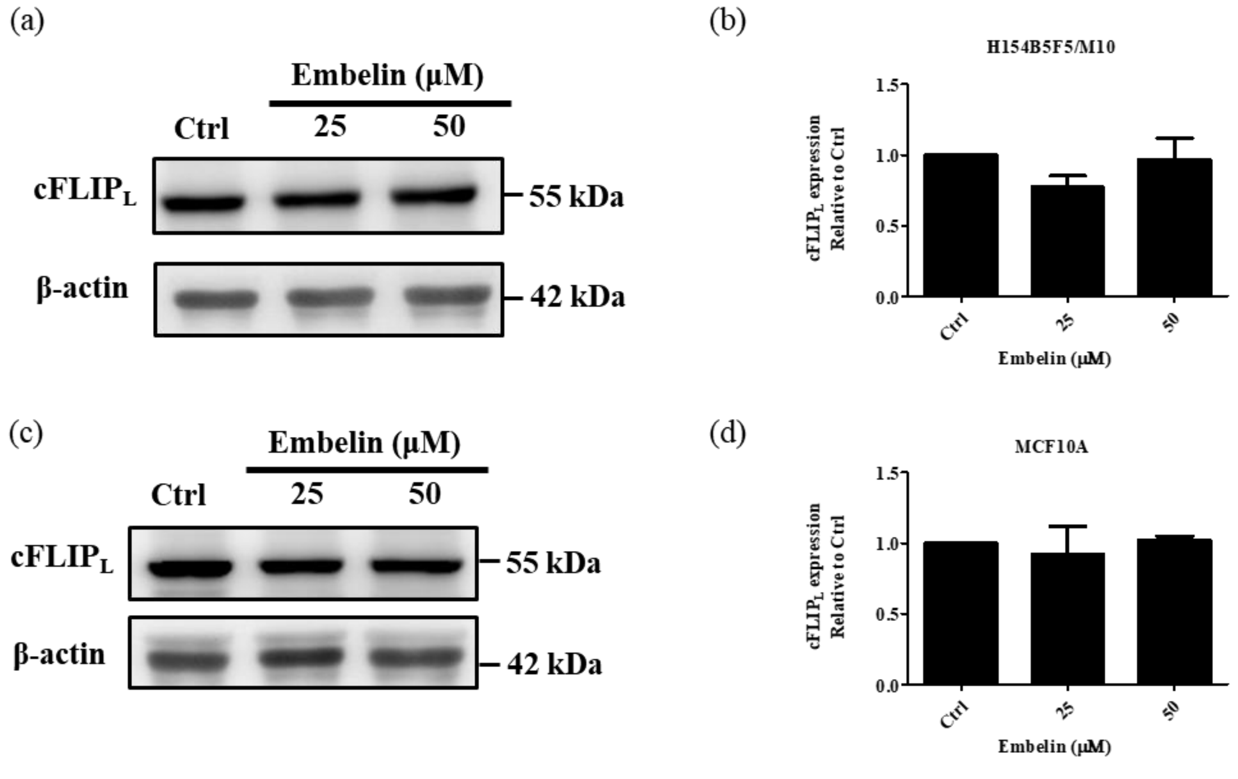

Supplementary Figure 2. Effects of IL-1β on cell viability and apoptosis of hUCMSCs.

hUCMSCs were treated with 100 ng/mL IL-1β for 24 hours. (a) The cell viability was detected by MTT assay. (b) hUCMSCs were stained by Annexin V/ propidium (PI) dye and the apoptosis rate was determined by flow cytometry.

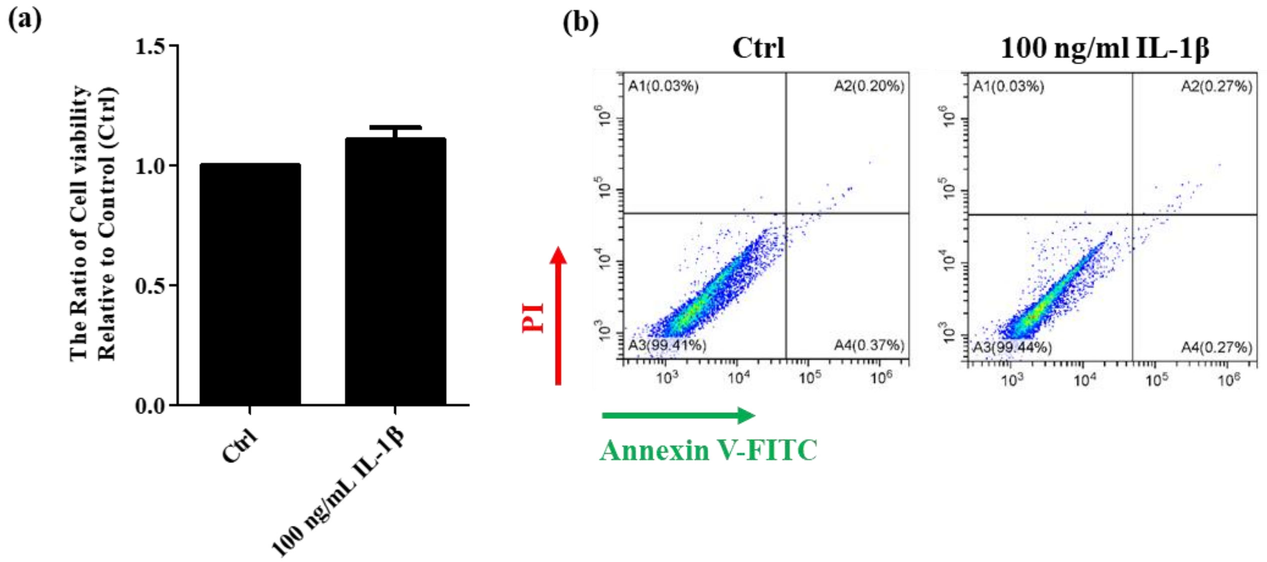

Supplementary Figure 3. Full- length Western blots.

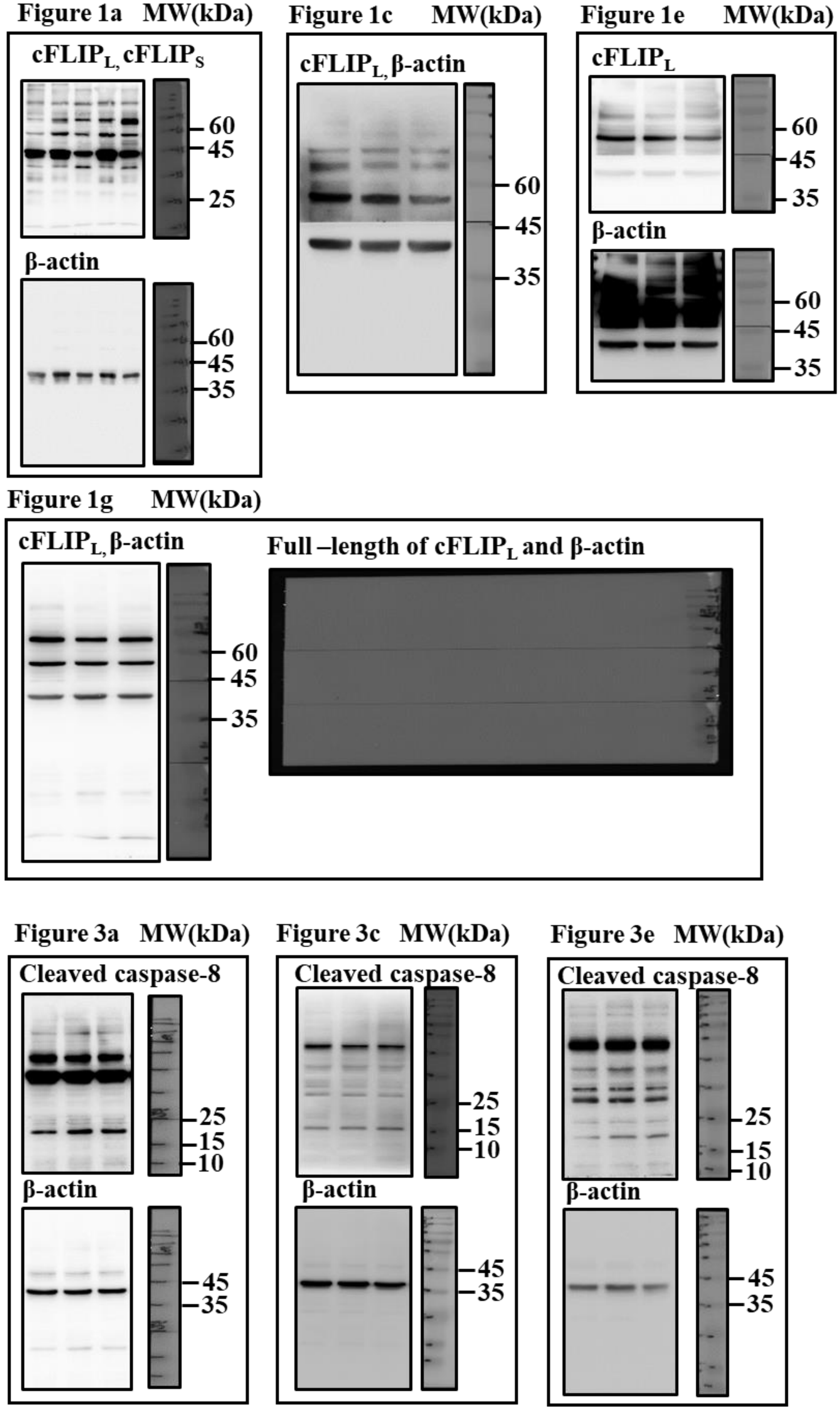

Figure 3g MW(kDa)

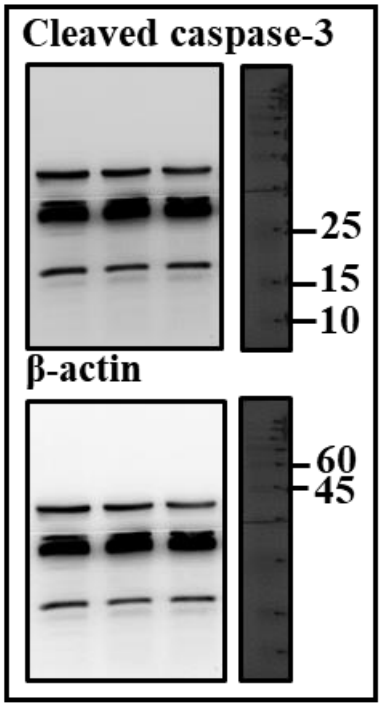

Figure 3i MW(kDa)

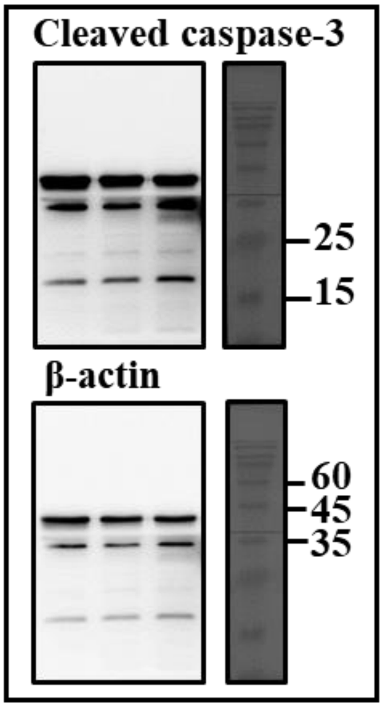

Figure 3k MW(kDa)

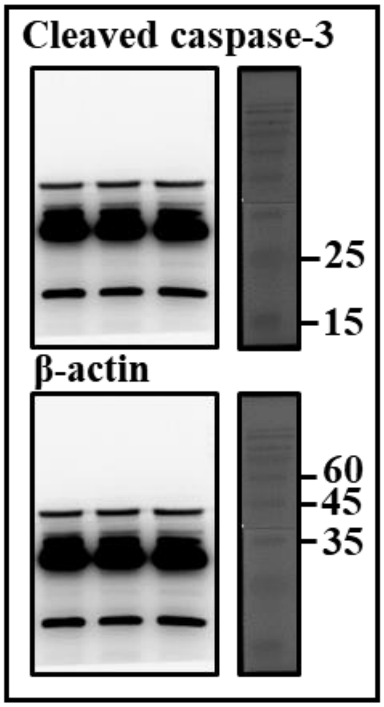

Figure 3m MW(kDa)

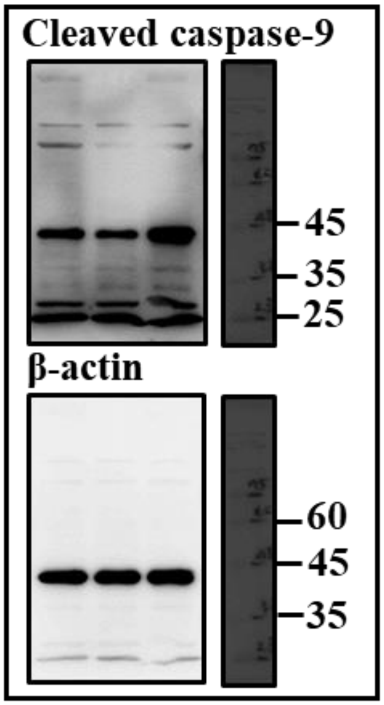

Figure 3o MW(kDa)

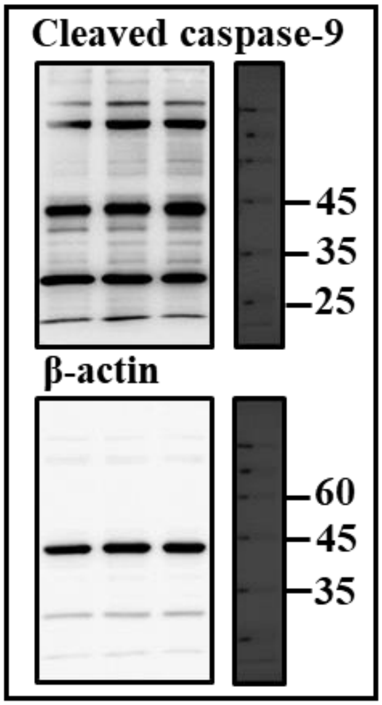

Figure 3q MW(kDa)

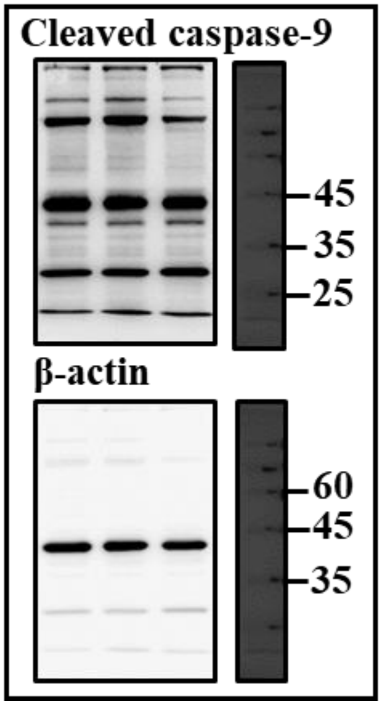

Figure 4a MW(kDa)

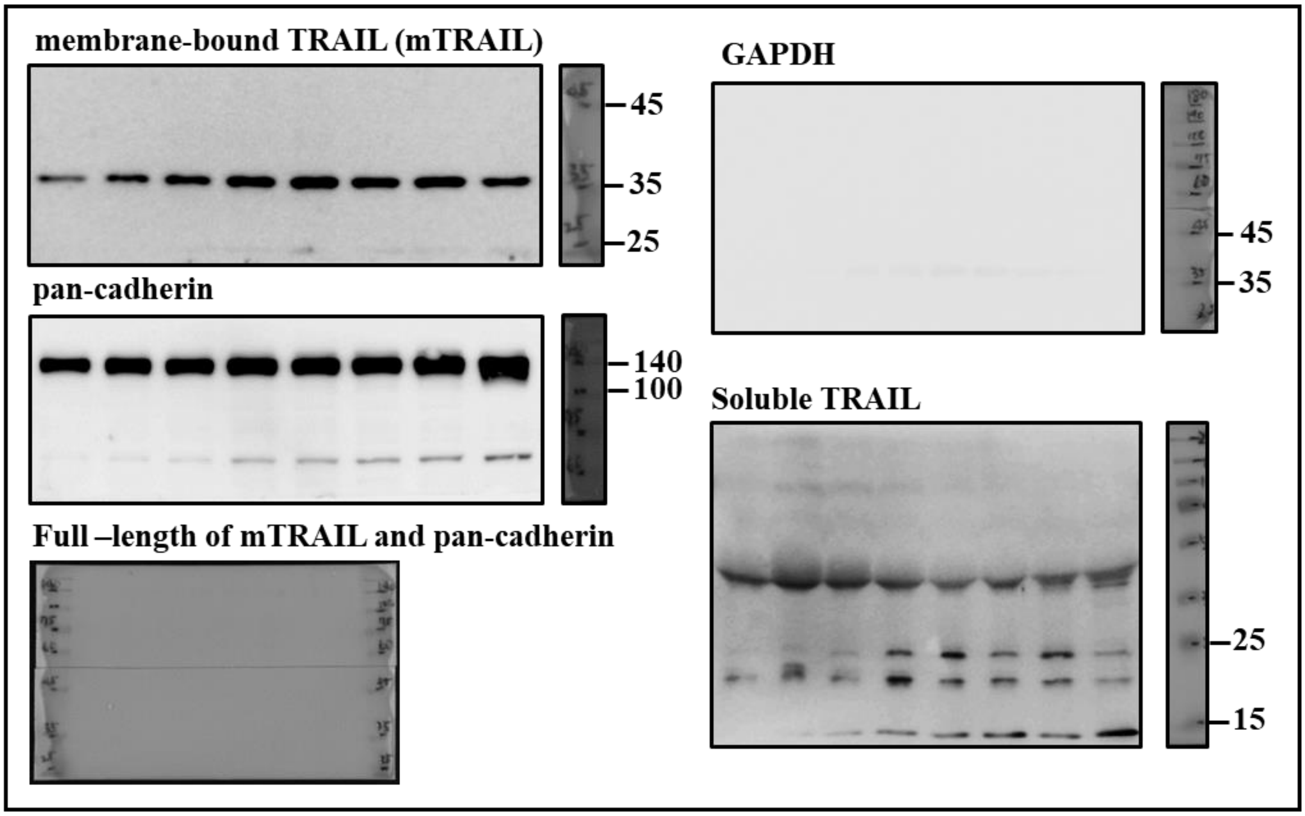

Supplementary figure 2a MW(kDa)

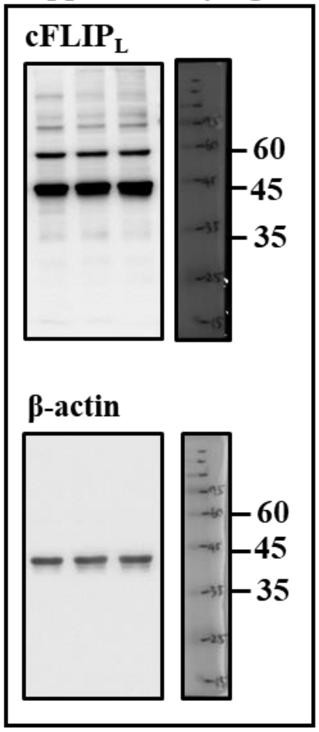

Supplementary figure 2c MW(kDa)

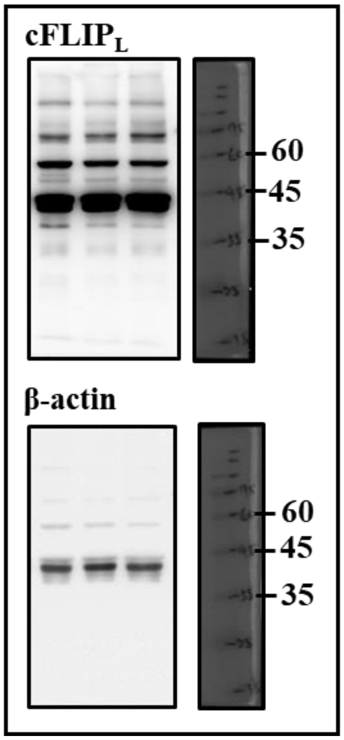

Supplement: Supplementary file 1 — Supplementary Information. [file 41598_2021_94006_MOESM1_ESM.pdf]
